# Identification of effector CEP112 that promotes the infection of necrotrophic *Alternaria solani*

**DOI:** 10.1186/s12870-022-03845-w

**Published:** 2022-09-29

**Authors:** Chen Wang, Dai Zhang, Jianing Cheng, Dongmei Zhao, Yang Pan, Qian Li, Jiehua Zhu, Zhihui Yang, Jinhui Wang

**Affiliations:** 1 College of Plant Protection, Hebei Agricultural University, Baoding, 071001 People’s Republic of China; 2 Technological Innovation Center for Biological Control of Crop Diseases and Insect Pests of Hebei Province, Baoding, 071001 People’s Republic of China

**Keywords:** *Alternaria solani*, Effector, Chlorosis, Senescence, Gene knockout, Pathogenicity

## Abstract

**Background:**

*Alternaria solani* is a typical necrotrophic pathogen that can cause severe early blight on Solanaceae crops and cause ring disease on plant leaves. Phytopathogens produce secretory effectors that regulate the host immune response and promote pathogenic infection. Effector proteins, as specialized secretions of host-infecting pathogens, play important roles in disrupting host defense systems. At present, the role of the effector secreted by *A. solani* during infection remains unclear. We report the identification and characterization of AsCEP112, an effector required for *A. solani* virulence.

**Result:**

The *AsCEP112* gene was screened from the transcriptome and genome of *A. solani* on the basis of typical effector signatures. Fluorescence quantification and transient expression analysis showed that the expression level of *AsCEP112* continued to increase during infection. The protein localized to the cell membrane of *Nicotiana benthamiana* and regulated senescence-related genes, resulting in the chlorosis of *N. benthamiana* and tomato leaves. Moreover, comparative analysis of AsCEP112 mutant obtained by homologous recombination with wild-type and revertant strains indicated that *AsCEP112* gene played an active role in regulating melanin formation and penetration in the pathogen. Deletion of *AsCEP112* also reduced the pathogenicity of HWC-168.

**Conclusion:**

Our findings demonstrate that AsCEP112 was an important effector protein that targeted host cell membranes. AsCEP112 regulateed host senescence-related genes to control host leaf senescence and chlorosis, and contribute to pathogen virulence.

**Supplementary Information:**

The online version contains supplementary material available at 10.1186/s12870-022-03845-w.

## Introduction

*Alternaria solani*, a typical necrotrophic pathogen, is the main causal agent of early blight, which is a destructive foliar disease that affects potato, tomato and other cultivated Solanum species [1, 2]. The typical disease symptoms are dark brown to black lesions with concentric rings on the leaves, which result in leaf browning and drop in severe cases. During the infection process, *A. solani* produces germ tubes to penetrate host tissues or directly invades from stomata or wounds adjacent to epidermal cells. It secretes metabolites to digest host cell inclusions, thereby causing infection [3]. Similarly, the metabolites of *A. solani* also reduces the photosynthetic rate in infected leaves by inhibiting the photosystem II activity and reducing the chlorophyll and other photosynthetic pigment contents, resulting in a general reduction in plant growth and a decline in yield [4, 5].

Necrotrophic pathogens can directly kill host tissues, and they obtain nutrients mainly from dead cells. Cell death is also an important early-stage signal of the successful infection by necrotrophic pathogens [6–8]. Necrotrophic pathogens may thrive by subverting the resistance mechanisms acquired by plants to combat other pathogens [9, 10]. They can be divided into two categories on the basis of their host range. One category contains pathogens that are host-specific necrotrophs, which usually produce host-selective toxins [11, 12]. The other category contains pathogens, including *A. solani*, that are broad host-range necrotrophs. Compared with biotrophs, the interaction mode of necrotrophs with a large range of host plants remains to be explored. In plant defense responses to attacking pathogens, salicylic acid (SA) dependent defenses act against biotrophs, and jasmonic acid (JA) and ethylene (ET) dependent responses act against necrotrophs [13, 14]. However, an increasing number of studies have found that SA signaling, and not JA signaling, is required for host defenses against certain necrotrophic pathogens [15, 16].

Many necrotrophs enhance pathogenicity using plant defense responses, and they induce host-cell death by secreting phytotoxic secondary metabolites [17, 18]. Some low molecular weight metabolites and secreted proteins are the basic determinants of pathogenicity or virulence [19]. Effectors enable successful pathogens to overcome MAMP-triggered immunity, leading to effector-triggered susceptibility. These effectors can be recognized by intracellular receptors (R proteins) to activate effector-triggered immunity (ETI), a major source of qualitative resistance to biotrophs [20–22]. However, the effectors are co-opted by necrotrophs to promote disease [23]. Until now, no effector had been cloned from *A. solani*. Thus, the identification of effector proteins is of great significance for understanding the pathogenic mechanism of necrotrophic *A. solani*.

Based on the transcriptome and genome results of *Alternaria solani*, we selected a novel effector, AsCEP112, which localized to the host cell membrane and was highly expressed during infection. Transient expression assays in *Nicotiana benthamiana* and tomato showed that AsCEP112 regulated host senescence-related genes to promote host chlorosis. Moreover, we demonstrated that the deletion of the *AsCEP112* gene did not affect pathogen vegetative growth or development, but it reduced the melanin content and penetrability. It also significantly reduced virulence. These results suggest that AsCEP112 is critical for the virulence of *A. solani* and provide insights into the molecular interactions between *A. solani* and its host.

## Materials and methods

### Plants, fungal and bacterial strains.

The wild-type strain HWC-168 of *A. solani* was collected from leaves infected with potato early blight in Weichang County, Hebei Province, China. All the plants were grown on the campus of Hebei Agricultural University (Baoding, China). *Nicotiana benthamiana* and tomato were grown in a greenhouse at 23 ± 1 °C with a 16-h light period. Potato ‘Favorita’ was grown in a greenhouse at 20 ± 1 °C. The WT *A. solani* HWC-168 strain was cultured on Potato Dextrose Agar (PDA) medium in the dark at 25 °C. *Escherichia coli* strain DH5α was cultured in Luria–Bertani (LB) medium at 37 °C and used for plasmid amplification. *Agrobacterium tumefaciens* strain EHA105 was cultured in LB medium at 28 °C and used for *Agrobacterium*-mediated transient expression in plant leaves.

### Sequence analysis

The Online tools SignalP 5.0 Server (http://www.cbs.dtu.dk/services/SignalP/), SMART (http://smart.embl-heidelberg.de/) and ProParam (https://web.expasy.org/protparam/) were used to predict protein signal peptides, structural domains, molecular masses and theoretical isoelectric points.

### RNA isolation and cDNA synthesis

In this study, the hyphae of *A. solani* and the leaves of potato infected at different time points were selected for gene expression analyses. The hyphal and plant total RNAs were extracted using an Easy Pure Plant RNA Kit (TransGen Biotech, Beijing, China) in accordance with the manufacturer’s instructions. The total RNA concentrations were determined by spectrophotometric analysis and adjusted to be uniform. Using the TransScript One-Step gDNA Removal and cDNA Synthesis SuperMix (TransGen Biotech), 400 ng to 500 ng of total RNA was used to synthesize first-strand cDNA.

### Quantitative real-time PCR analysis

Based on previous studies on *A. solani*, we selected a relatively stable housekeeping gene "*Actin*" as an internal reference gene [24, 25]. Specific primers for *Actin* and *AsCEP112* were designed using the National Center for Biotechnology Information (NCBI) database (https://www.ncbi.nlm.nih.gov/) (Supplementary Table S2). The SEN4, SAG12 and DHAR1 sequences were obtained from Uniport (https://www.uniprot.org/), and Primer 3 Plus (https://www.primer3plus.com/) was used to design specific primers for each gene. PP2A was selected as a housekeeping gene in N. benthamiana [26]. The cDNAs of the hyphae growing on potato and *N. benthamiana* leaves were serial diluted to detect primer specificity. The qRT-PCR was performed using the Bio-Rad CFX384 Touch Real-Time System (Bio-Rad, USA), and the total reaction volume was 20 μL, containing 2 μL gene specific primers, 6 μL of cDNA and 10 μL of 2 × Magic SYBR Mixture (CWBIO, Jiangsu, China). The qRT-PCR was performed under the following conditions: 95 °C for 30 s, followed by 40 cycles at 95 °C for 5 s and 59 °C for 30 s. The melting curve analysis was performed at 95 °C for 15 s, 60 °C for 1 min and 95 °C for 15 s, with a final step at 50 °C for an additional 30 s. The threshold cycle (CT) values were used to determine the fold change in transcript accumulation with the log_2_((1 + E1)^△Ct1(ControlSample)^/(1 + E2)^△Ct2(Control−Sample)^) method [27]. Six biological and two technical replicates were performed for each sample in all the qRT-PCR reactions.

### Subcellular localization and hypersensitive response assay

The transient expression of *AsCEP112* was performed using an *Agrobacterium*-mediated transient gene expression system [28, 29]. The full-length open reading frame (referred to as FL), or the truncated coding region without the signal peptide sequence but with an engineered ATG start codon (referred to as NSP), of *AsCEP112* was PCR amplified using specific primers and integrated independently into the binary vector pCAMBIA1301::EGFP at a position downstream of the CaMV35S promoter. Then, the fusion vectors were transformed independently into the *A. tumefaciens* strain EHA105 [30]. The amplification product and pCAMBIA1301 vector digested with restriction enzymes SalI and BamHI (TaKaRa, Beijing, China) were ligated using a pEASY-Uni Seamless Cloning and Assembly Kit (TransGen Biotech).

INF1, an elicitin secreted by *Phytophthora infestans*, which induces defense responses including the hypersensitive response (HR), was used as the positive control in Solanaceae family infiltration tests, specifically *Nicotiana* species [31]. Recombinant *Agrobacterium* was cultured at 28 °C and resuspended in an infiltration medium containing 10 mmol L^−1^ MgCl_2_, 10 mmol L^−1^ 2-(N-morpholino) ethanesulfonic acid (pH 5.6) and 200 μmol L^−1^ acetosyringone. The recombinant strains were tested on *N. benthamiana* leaves at an optical density (OD)_600_ of 0.6–0.8. Different infiltration groups were injected into the leaves of *N. benthamiana*. At 72 h after incubation, the treated leaves were collected, and subcellular *AsCEP112* localization was observed under a confocal laser microscope (TCS SP8, Leica, Germany) with excitation at 488 nm and emission at 507 nm.

For the detection of HR, the strains expressing the AsCEP112 protein were infiltrated into the right side of each leaf, and the empty vector (EV) was infiltrated into the left side. The *N. benthamiana* and tomato were cultured for 5 days at 23 °C until the disease symptom appeared. Whole *N. benthamiana* and tomato leaves were colored using a DAB Kit (CWBIO) at room temperature. After 12 h, the leaves were discolored by boiling in 95% ethanol for 30 min and photographed. Each treatment was replicated six times.

### Signal peptide secretion assay

A yeast secretion system was used to validate the function of predicted signal peptides. The signal peptide of AsCEP112 was cloned into vector pSUC2 using specific primers, and the recombinant plasmid was transformed into yeast strain YTK12. The positive colonies were screened on a CMD − W medium (0.075% tryptophan dropout supplement, 0.67% yeast nitrogen base without amino acids, 2% sucrose, 0.1% glucose and 2% agar). To test for invertase secretion, successfully transformed yeast strains were grown on YPRA agar (1% yeast extract, 2% peptone, 2% raffinose, 2 mg/mL antimycin A and 2% agar).

### Deletion and restoration of *AsCEP112* in *A. solani*

A gene knockout strategy with homologous recombination technology was used for *A. solani*. The hygromycin B resistance gene (*Hyg*) was used to mark the knockout *AsCEP112* gene. Using specific primers, approximately 1-kb upstream and downstream fragments from the WT HWC-168 genomic DNA and the split hygromycin B resistant gene fragments from the pUC-HYG plasmid were amplified for collective transformation into the pUC19 vector using restriction enzymes EcoRI and BamHI and the pEASY-Uni Seamless Cloning and Assembly Kit. The PCR products were purified from the gels using a SanPrep Column DNA Gel Extraction Kit (Sangon Biotech, Shanghai, China). Mixtures of upstream- and downstream-hygromycin fusion fragments were used for transforming protoplasts of *A. solani*.

The WT HWC-168 hyphae were inoculated into 200-mL flasks containing 100 mL Potato Dextrose Broth (PDB) medium, incubated at 25 °C and shaken at 120 rpm min^−1^ for 36 h. The mycelia were filtered through three layers of gauze and rinsed 2–3 times thoroughly with 0.7 mol L^−1^ NaCl solution. The hyphae were transferred to a 50-mL centrifuge tube using tweezers, and the protoplasts were digested with a mixture of enzymes, 0.1 mg of snailase (Solarbio, Beijing, China), 0.1 mg of driselase (Biotopped, Beijing, China) and 0.1 mg of lysing enzyme (SIGMA, Shanghai, China) in 10 mL of 0.7 mol L^–1^ NaCl. The protoplasts were resuspended in STC buffer (1.168 M D-Sorbitol, 10 mM Tris–HCl and 4.96 mM CaCl_2_) and the fusion fragments mixtures were added to the PTC buffer (15 mM PEG4000, 10 mM Tris–HCl and 10 mM CaCl_2_). The transformants were screened on Regeneration PDA medium containing ampicillin (50 μg mL^–1^) and hygromycin (50 μg mL^–1^), and PCR was performed using specific primers to confirm the transformants.

After successfully obtaining the *AsCEP112* gene mutant strain, the mutant strain was used to produce *AsCEP112* protoplasts. The *AsCEP112-*FL gene was amplified with specific primers, and it and the vector KN containing the neomycin resistance gene (Neo) were digested with restriction enzymes EcoRI and BamHI. The fragments were ligated using the pEASY-Uni Seamless Cloning and Assembly Kit, and the constructed plasmid was transformed into *AsCEP112* mutant protoplasts using PEG-mediated protoplast transformation technology. Finally, neomycin resistance was used to screen for revertant strains.

### Melanin, penetration and pathogenicity tests

The colonies of the WT, *AsCEP112* mutant and revertant strains having diameters greater than 5 mm were inoculated independently into 350-mL flasks containing 100 mL PDB medium, incubated at 25 °C and shaken at 150 rpm min^−1^ for 5 d. Then, the media of the WT, *AsCEP112* mutant and revertant strains were filtered through gauze, and the absorbance levels of the solutions were measured at 400 nm.

Sterile cellophane was spread on the PDA medium, and then, the WT, deletion mutant and revertant strains were placed on the PDA medium and incubated at 25 °C for 3 d in the dark. Then, the cellophane was removed and the strains were cultured for 4–5 d.

*Alternaria solani* strain HWC-168 was inoculated into tomato agar medium. After the hyphae were overgrown, mechanical damage was performed, and sporulation was stimulated by ultraviolet light for 15 min. Spore suspensions of *A. solani* WT, *AsCEP112* mutant and revertant strains were prepared. The final spore concentrations were adjusted to 10^5^ mL^−1^ per suspension. Five-week-old potato leaves were sterilized with 70% alcohol, washed with sterile water, and then, the petioles were inserted into water agar medium. Approximately 20 µL of each suspension was inoculated into potato leaves in vitro. The leaves were incubated at 25 °C under a 16-h photoperiod. The accumulation of H_2_O_2_ was detected by DAB staining [32]. In vitro potato leaves were harvested at 5 dpi and immersed in DAB solution (CWBIO) at room temperature. After 12 h, the leaves were discolored and photographed by boiling them in 95% ethanol for 30 min. The tests were conducted in three replicates.

## Results

### Bioinformatics identification of AsCEP112

By comparing the genome and transcriptome information of *A. solani*, we identified the *AsCEP112* gene, which had effector characteristics, and analyzed its function. The signal peptide of the *AsCEP112* gene correponded to amino acids (aa) 1 to 18, and no signal domain was identified using online tools (Supplementary Table S1). To determine the phylogenetic relationship of the identified AsCEP112, the amino acid sequences of complete proteins were aligned in NCBI using MEGA, and a Maximum-Likelihood phylogenetic tree was constructed (Supplementary Figure S1). However, no CEP112 homologs were found in genera other than *Alternaria*. The results indicated that the *AsCEP112* sequence was more closely related to other Alternaria spp. Thus, it may have formed by vertical gene transfer in *A. solani*.

### Expression patterns of AsCEP112 in the infection of *A. solani*

**Fig. 1:**
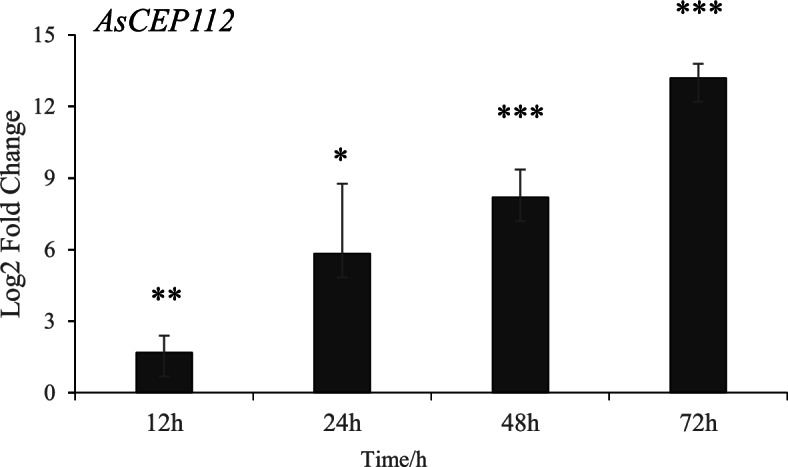
Expression patterns of the *AsCEP112* gene during the infection of potato leaves. The expression level at the hyphal stage was used as a control to analyze the specific expression at the infection stage. Actin expression was used as an internal reference for normalizing within the samples. Student’s t-test was used as a significance test of differences (*n* = 6). *, indicates a difference (0.01 < *p* < 0.05) compared with the control (hyphal stage); **, indicates a significant difference (0.001 < *p* < 0.01) compared with the control; ***, indicates an extremely significant difference (*p* < 0.001) compared with the control. Bars represent standard deviations (SDs)

To identify the role of *AsCEP112* in the infection of *A. solani*, its temporal expression pattern was assessed by qRT-PCR of infected potato leaves compared with mycelia. The expression of AsCEP112 in the infection stage continued to increase compared with in the hyphal stage and the log_2_ fold change of *AsCEP112* expression was up-regulated by 13.18 fold at 72 hpi (Fig. 1).

### Subcellular localization of AsCEP112 in *N. benthamiana*

SignalP 5.0 predicted the N-terminal 18 amino acids of AsCEP112 as a specific signal peptide sequence [33]. The secretory function of the putative signal peptide was confirmed using a yeast invertase secretion assay. When the predicted signal peptide of AsCEP112 was fused to the yeast invertase sequence in the vector pSUC2, it mediated the complementation of yeast YTK12 mutant strains (invertase-deficient) grown on raffinose or YPRA agar. These results indicated that the signal peptide of AsCEP112 had a secretory function (Supplementary Fig. S2).

**Fig. 2:**
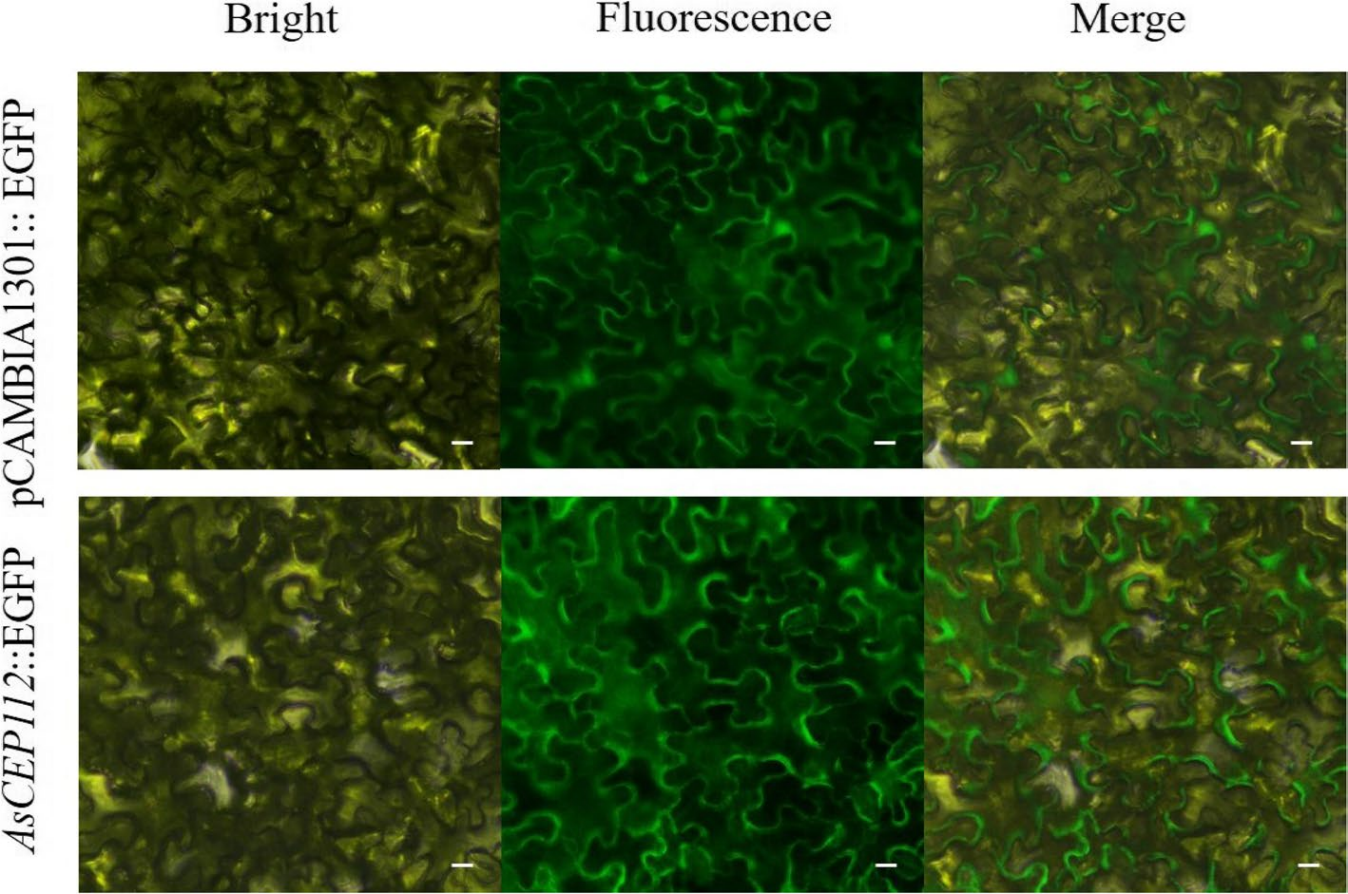
Subcellular localization of *AsCEP112* proteins in *N. benthamiana*. The *Agrobacterium* strain EHA105 containing the pCAMBIA1301 vector, as the control, and the *AsCEP112* gene were independently transiently expressed in *N. benthamiana* leaves. Bar = 20 μm

Secreted effector proteins enter different host cell locations depending on their roles. Effector proteins regulate functions by targeting different organelles and ultimately destroy host defense signaling, which increases susceptibility [34]. When fungal effectors are secreted, they function either in the apoplastic spaces or inside host cells after translocation. Therefore, to detect the localization of AsCEP112 in the host, we performed a subcellular localization assay with the green fluorescent protein (GFP) fusion. It indicated that the overexpressed *AsCEP112*-NSP (lacking the N-terminal SP) in *N. benthamiana* eventually acted on the membranes of host cells (Fig. 2).

### AsCEP112 promotes chlorosis of *N. benthamiana* and Tomato

Fungal pathogens manipulate host immune responses to promote infection by secreting specific effector proteins. To determine whether AsCEP112 had the function of inducing host-cell death, suppressing host-immune response or promoting host-cell death, we performed transient expression assays in *N. benthamiana*. As the positive control, INF1, which induces programmed cell death in *N. benthamiana*, was used.

**Fig. 3:**
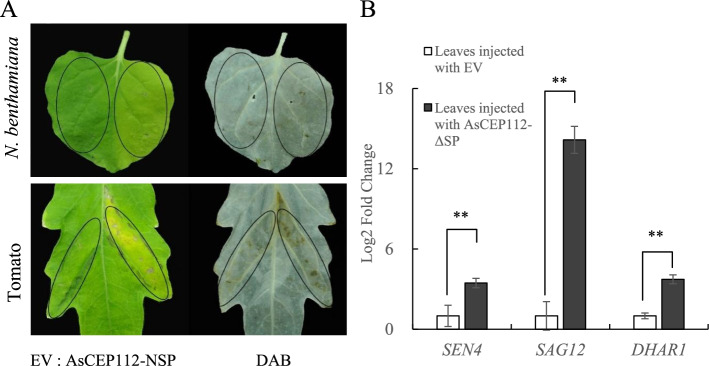
*Agrobacterium* containing AsCEP112 effector pro*mo*tes senescence of *N. bethamiana* and tomato leaves. **A** *Nicotiana benthamiana* and tomato leaves were infiltrated by control (EV; left, leaf tip to petiole direction) and pCAMBIA1301 *Agrobacterium* containing *AsCEP112*-NSP gene (right, leaf tip to petiole direction). **B** Relative mRNA levels of *SEN4*, *SAG12* and *DHAR1*. Expression levels of senescence- and oxidative stress-associated genes were examined in *N. benthamiana* (EV and *AsCEP112*-NSP) and coi1 shoots 4 days after injection with *Agrobacterium*

When AsCEP112 (FL and NSP) was injected alone, the leaves showed no symptoms of cell death. When INF1 was co-injected with AsCEP112 (FL and NSP), host immune responses were not suppressed (Supplementary Fig. S3). In addition, we determined whether *AsCEP112* promoted infection. The pCAMBIA1301 vector was used as a negative control. The *N. benthamiana* leaves infiltrated with *Agrobacterium AsCEP112*-NSP alone showed chlorotic symptoms. At the same time, we also repeated this experiment in tomato plant leaves, and found that *Agrobacterium AsCEP112*-NSP also caused chlorotic symptoms on tomato leaves. In addition, we investigated the ability of AsCEP112-NSP to activate hydrogen peroxide (H_2_O_2_) in *N. benthamiana* and tomato leaves. Compared with the control, H_2_O_2_ was activated by AsCEP112-NSP at 5 dpi (Fig. 3).

Many genes that regulate plant growth during senescence are defined as senescence-related genes [35]. The transcription levels of three senescence-related genes (*SEN4*, *SAG12* and *DHAR1*) were independently determined in the *N. benthamiana* leaves infiltrated with EV and AsCEP112-NSP. After treatment with ASCEP112-NSP, the expression levels of senescence-related genes were significantly up-regulated compared with after the EV treatment. *SAG12* expression was upregulated the most, being 14.16-fold higher than in the EV treatment. These results suggested that AsCEP112 induces plant chlorotic symptoms by regulating senescence-related genes.

### Construction and confirmation of *AsCEP112* deletion and revertant strains

**Fig. 4:**
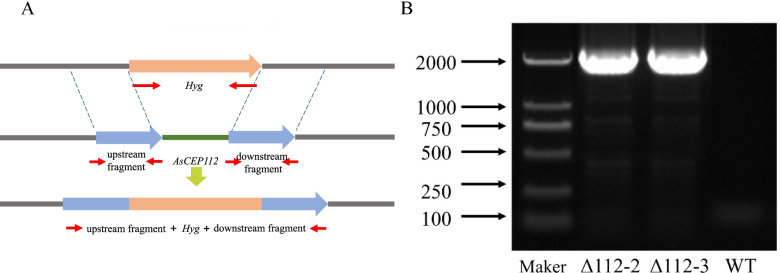
*AsCEP112* gene replacement and RT-PCR screening of mutants. **A** Diagram of the homologous recombination of *AsCEP112* and *Hyg* genes. **B** Verification of the *Hyg* gene in the genomes of WT and *AsCEP112* mutant strains

Homologous recombination technology was used to obtain *AsCEP112* deletion mutants through PEG-mediated transformation of protoplasts. Hyg-specific primers were used to verify the mutant strain by RT-PCR. A band was detected in the transformants, but no band was detected in the WT strains (Fig. 4). Thus, *AsCEP112* was replaced by a single copy of *Hyg* in the genomic DNA of *A. solani*. In this study, two knockout mutants of *AsCEP112* were obtained.

In the same manner, the *Hyg* gene in each *AsCEP112* deletion strain was replaced with the *AsCEP112* gene to obtain two corresponding revertant strains. *AsCEP112* gene-specific primers were used to detect mutant strains (Supplementary Fig. S4).

### *AsCEP112* affects colony melanin and penetration, but not the growth and sporulation rates

To verify the role of *AsCEP112* in the growth of *A. solani*, we compared the morphology, growth rate, sporulation rate and penetration of the WT, *AsCEP112* mutant and revertant strains. The colony growth and sporulation rates of the WT, *AsCEP112* mutant and revertant strains were roughly the same (Supplementary Fig. S5).

**Fig. 5:**
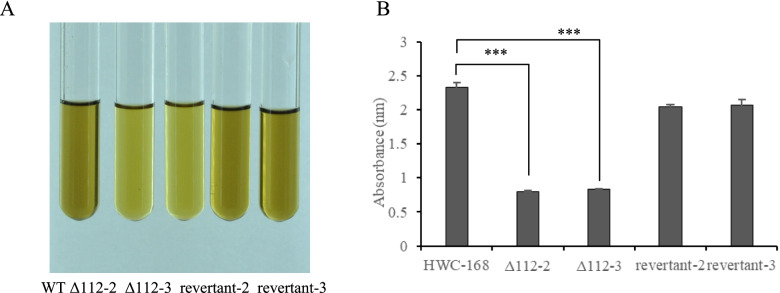
Determination of the phenotypes and melanin levels of the wild-type, *AsCEP112* mutant and revertant strains. **A** The solutions of wild-type, *AsCEP112* mutant and revertant strains for the determination of the melanin contents. **B** The absorbance values of the wild-type, *AsCEP112* mutant and revertant strain solutions at 400 nm. *, indicates a difference (*p* < 0.05) compared with the control (HWC-168); ***, indicates an extremely significant difference (*p* < 0.001) compared with the control (HWC-168). Bars represent standard deviations (SDs)

The melanin area in the inner circles of the *AsCEP112* mutant strain colonies were smaller compared with those of the WT and revertant strains (Supplementary Fig. S6). Therefore, the melanin content of the WT, *AsCEP112* mutant and revertant strains were determined. The solutions of the *AsCEP112* mutant strains were discolored. After measuring the OD_400_ of the solutions, it was determined that the absorbance levels of the *AsCEP112* mutant strains decreased significantly compared with those of the other strains (Fig. 5).

**Fig. 6:**
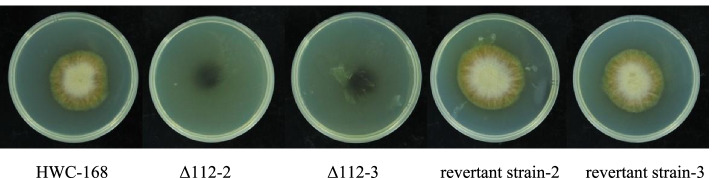
Penetration of the WT, *AsCEP112* mutant and revertant strains. The colony morphology of wild-type, *AsCEP112* mutant and revertant strains filtered through three layers of sterile cellophane

A comparison of the mechanical penetrations of the WT, *AsCEP112* mutant and revertant strains revealed that when three layers of cellophane were spread on the PDA medium, the cellophane was penetrated by the WT and the revertant strains. However, the *AsCEP112* mutant strains did not penetrate the cellophane although they left melanin on the PDA medium (Fig. 6). The results indicated that the absence of *AsCEP112* reduced the penetration capability of *A. solani*.

### Susceptibility of the *AsCEP112* knockout mutants to pathogenicity

**Fig. 7:**
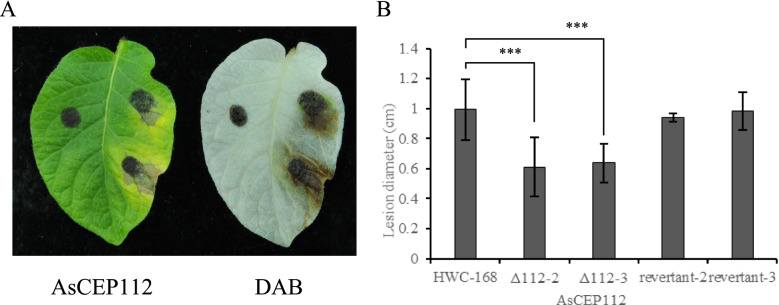
Detecting the pathogenicity of the *AsCEP112* gene. **A** The isolated potato leaves were inoculated with the spore suspensions of *AsCEP112* mutant (left, leaf tip to petiole direction), wild-type (upper right, leaf tip to petiole direction) and revertant (lower right, leaf tip to petiole direction) strains. **B** The diameters of lesions on potato leaves infected by the wild-type, mutant Δ112-2, mutant Δ112-3, revertant-2 and revertant-3 strains. ***, indicates an extremely significant difference (*p* < 0.001) compared with the control (HWC-168). Bars represent standard deviations (SDs)

To determine whether the deletion of *AsCEP112* contributed to the full pathogenicity of *A. solani*, the isolated leaves of potato were inoculated with the spore suspensions of the WT, AsCEP112 mutant and revertant strains. The average diameter of the lesions of the *AsCEP112* mutant strain was approximately 62.86% that of the WT strain. The average diameter of lesions of the WT strain was approximately 0.99 cm. The average diameters of lesions of the mutant strains were approximately 0.61 and 0.64 cm, and the average diameters of lesions of the revertant strains were approximately 0.94 and 0.98 cm. Compared with the WT and revertant strains, the *AsCEP112* mutant strains produced smaller lesion areas, and no chlorotic symptoms were obvious on the leaves. Moreover, after the leaves were stained with DAB solution, the accumulations of H_2_O_2_ at the inoculation sites of the *AsCEP112* mutant strains were less than those of the WT and revertant strains (Fig. 7). Thus, the deletion of *AsCEP112* caused a decrease in the pathogenicity of *A. solani* infecting potato leaves. The statistical analysis showed a significant difference (*p* < 0.001) in the sizes of the lesions produced by WT and mutant strains.

## Discussion

*Alternaria solani* is a kind of necrotrophic pathogen that can cause early blight disease of tomato, potato, tobacco, and many other vegetables and crops, and lead to huge losses in agricultural production [36]. The changes in the JA- and SA-dependent defenses pathways in the host during an *A. solani* infection are different from those that occur during common necrotroph infections [37]. To date, no in-depth studies on the factors released by *A. solani* during the potato infection process have been reported. Thus, it is urgent to explore the pathogenic mechanism of *Alternaria solani* involved in infecting potatoes.

Specific effectors are expressed during different pathogenic stages, indicating that specific effectors play critical roles in the corresponding infection stages [38]. The expression of the *AsCEP112* gene continued to increase for 72 h after *A. solani* infected the potato. The first 72 h of *A. solani* infection is also the most intense stage of host-cell damage. *Alternaria solani* invades host cells through hyphae by 8 hpi, and then destroys the host cells by 24 hpi. At 72 hpi, the host cells are basically eliminated [39]. The changes in host cells during the entire infection process corresponded to the changes in *AsCEP112* expression. Additionally, the expression levels of defense-related genes and defense-related enzymes (SOD, POD and PAL) in the host also increase significantly in 72 h, indicating that the substances secreted by *A. solani* cause serious damage to defense-related responses of the host [40]. The change in the expression of *AsCEP112* probably indicates that it plays a crucial pathogenic function in the *A. solani* infection process.

Phytopathogen effectors are usually overexpressed in plants to induce phenotypes, reflecting their virulence activity levels. Effectors with different functions may play roles in inhibiting host-cell death or inducing host-cell death during the infection [41]. *Sclerotinia sclerotiorum*, a typical necrotrophic pathogen, induces significant cell death by secreting the SsCP1 effector, and it inhibits the host immune responses by secreting the SsITL effector [42, 43]. Using the yeast signal trap assay system, we found that the AsCEP112 signal peptide was fused to the invertase gene, resulting in the secretion of invertase by yeast. This indicates that AsCEP112 was probably secreted into the extracellular space during host plant infection. For the transient expression of *AsCEP112*, there were no symptoms of inducing or inhibiting cell death. The expression symptoms and fluorescence quantitative detection of AsCEP112 in *N. benthamiana* and tomato leaves showed that AsCEP112 promoted leaf chlorosis by regulating host senescence-related genes. As a main host defense line against pathogens, the cell membrane has many recognition receptors, including receptor-like kinases and proteins. The pattern recognition receptors located on the cell membrane play an important role in this process [44, 45]. The localization of *AsCEP112* on the cell membrane indicated that *AsCEP112* tends to mimic pathogen-associated molecular patterns rather than specific domains. The disruption of an important defense pathway between plant cell membranes and chloroplasts ultimately disturbs SA-dependent defenses, and intact SA signaling is required for potato defenses against the necrotrophic pathogen *A. solani* [37, 46]. These results further strengthen the research value of the *AsCEP112* gene located on the cell membrane.

Melanin is ubiquitous in fungi, and it greatly improves the tolerance of fungi to the external environment, including ultraviolet radiation, enzymatic hydrolysis and extreme temperatures [47, 48]. Although the production of melanin is not necessary for pathogenic fungi, it has a protective function against external stress [49–51]. The deletion of *AsCEP112* reduced the melanin areas of the *A. solani* mutant strains. In addition, the disease incidence on detached leaves of inoculated potato showed that the absence of *AsCEP112* also decreased the incidence area. CfEC92 of *Colletotrichum fructicola*, F12 of *Fusarium graminearum* and AvrPtoB of *Pseudomona* all play important roles in promoting the virulence of the pathogen itself [52–54]. The transient expression of AsCEP112 and the determination of the phenotype and pathogenicity of the AsCEP112 mutant strain, confirmed the importance of *AsCEP112* in the *A. solani* infection process. It confirmed that we should further study the pathogenic mechanism of *AsCEP112* in host plants.

## Conclusion

In summary, we investigated gene functions of AsCEP112, a candidate effector selected from *A. solani.* The *AsCEP112* gene was highly expressed during potato infection and promoted host leaf chlorosis by regulating host senescence-related genes. The AsCEP112 mutant strains reduced melanin production, penetration and ultimately host virulence. However, the molecular mechanism of AsCEP112 in the host remains unclear. We will explore further the role of chlorosis caused by AsCEP112 in infection and possible targets in the potato host to elucidate the molecular basis of the interaction between this necrotrophic pathogen and its host.

## Supplementary Information

**Additional file 1:** **Table S1.** Bioinformatics-based identification of the *AsCEP112* protein. **Figure S1.** Detection of AsCEP112 Protein Signal Peptide. **Figure S3.** The signal peptide (SP) of AsCEP112 is functional. The validation of the function of AsCEP112SP with yeast signal trap assay. The YTK12 yeast strain containing pSUC2 is able to grow on a CMD−W medium without tryptophan, but not on YPRAA medium. AsCEP112SP can grow on both CMD−W and YPRAA media. The SP of Avr1b was used as positive control. **Figure S4.** Transient expression of AsCEP112 in N. *benthamiana* leaves. The upper left and upper right corners of the leaf were injected with control (EV) and AsCEP112 (FL/NSP), respectively. The lower left and right corners were respectively injected with INF1 and AsCEP112 (FL/NSP) coupled with INF1. **Figure S5. Figure S6.** The colony areas and growth radii of the WT, AsCEP112 mutant and revertant strains. **Figure S7.** Determination of the phenotypes of the wild-type, AsCEP112 mutant and revertant strains. The colony phenotypes of *Alternaria solani* wild-type (left 1), AsCEP112 mutant (middle 2, 3) and revertant (right 4, 5) strains cultured on PDA medium for 7 d at 25°C in the dark. **Figure S8.** Pathogenicity detection of AsCEP112 gene. The isolated potato leaves were inoculated with the spore suspensions of AsCEP112 mutant strains (left, leaf tip to petiole direction), wild-type strains (upper right, leaf tip to petiole direction) and revertant strains (lower right, leaf tip to petiole direction). **Figure S9.** Full-length gel of Figure S5. The red line is the intercepted part. **Figure S10.** Subcellular localization of AsCEP112 proteins in N. benthamiana. The Agrobacterium strain EHA105 containing the pCAMBIA1301 vector, as the control, and the AsCEP112 gene were independently transiently expressed in N. benthamiana leaves. Bar = 20 μm. **Figure S11.** Full-length gel of Figure 4B. The red box is the intercepted part. **Figure S12.** Subcellular localization of AsCEP112 proteins in N. *benthamiana*. All images are AsCEP112.

**Additional file 2.** 

**Additional file 3.** 

**Additional file 4.** 

## Data Availability

All datasets generated for this study are included in the manuscript/Supplementary Files. (Accession number of gene sequence: NCBI OM735616) (https://www.ncbi.nlm.nih.gov/).
