# Supplementary material for: Identification of effector CEP112 that promotes the infection of necrotrophic Alternaria solani
Source: BMC Plant Biol. 2022 Sep 29;22:466. doi: 10.1186/s12870-022-03845-w (PMC9520946; doi:10.1186/s12870-022-03845-w)
Supplement: Supplementary file 1 — Additional file 1: Table S1. Bioinformatics-based identification of the AsCEP112 protein. Figure S1. Detection of AsCEP112 Protein Signal Peptide. Figure S3. The signal peptide (SP) of AsCEP112 is functional. The validation of the function of AsCEP112SP with yeast signal trap assay. The YTK12 yeast strain containing pSUC2 is able to grow on a CMD−W medium without tryptophan, but not on YPRAA medium. AsCEP112SP can grow on both CMD−W and YPRAA media. The SP of Avr1b was used as positive control. Figure S4. Transient expression of AsCEP112 in N. benthamiana leaves. The upper left and upper right corners of the leaf were injected with control (EV) and AsCEP112 (FL/NSP), respectively. The lower left and right corners were respectively injected with INF1 and AsCEP112 (FL/NSP) coupled with INF1. Figure S5. Figure S6. The colony areas and growth radii of the WT, AsCEP112 mutant and revertant strains. Figure S7. Determination of the phenotypes of the wild-type, AsCEP112 mutant and revertant strains. The colony phenotypes of Alternaria solani wild-type (left 1), AsCEP112 mutant (middle 2, 3) and revertant (right 4, 5) strains cultured on PDA medium for 7 d at 25°C in the dark. Figure S8. Pathogenicity detection of AsCEP112 gene. The isolated potato leaves were inoculated with the spore suspensions of AsCEP112 mutant strains (left, leaf tip to petiole direction), wild-type strains (upper right, leaf tip to petiole direction) and revertant strains (lower right, leaf tip to petiole direction). Figure S9. Full-length gel of Figure S5. The red line is the intercepted part. Figure S10. Subcellular localization of AsCEP112 proteins in N. benthamiana. The Agrobacterium strain EHA105 containing the pCAMBIA1301 vector, as the control, and the AsCEP112 gene were independently transiently expressed in N. benthamiana leaves. Bar = 20 μm. Figure S11. Full-length gel of Figure 4B. The red box is the intercepted part. Figure S12. Subcellular localization of AsCEP112 proteins in N. benthamia [file 12870_2022_3845_MOESM1_ESM.docx]

Supplementary Material

# Table S1

| Table S1 Bioinformatics-based identification of the AsCEP112 protein | | | | | | | | | |  |
| --- | --- | --- | --- | --- | --- | --- | --- | --- | --- | --- |
| **Gene name** | **cDNA(bp)** | **amino acids** | **Singal peptide** | **Domain** | **Molecular weight(kDa)** | **pI** | **Instability index** | **Aliphatic index** | **GRAVY** |  |
|  |  |  |  |  |  |  |  |  |  |  |
| *AsCEP112* | 414 | 137 | 1-18 aa | 0 | 14.61 | 4.34 | 26.56 | 69.93 | -0.08 |  |

# Figure S1


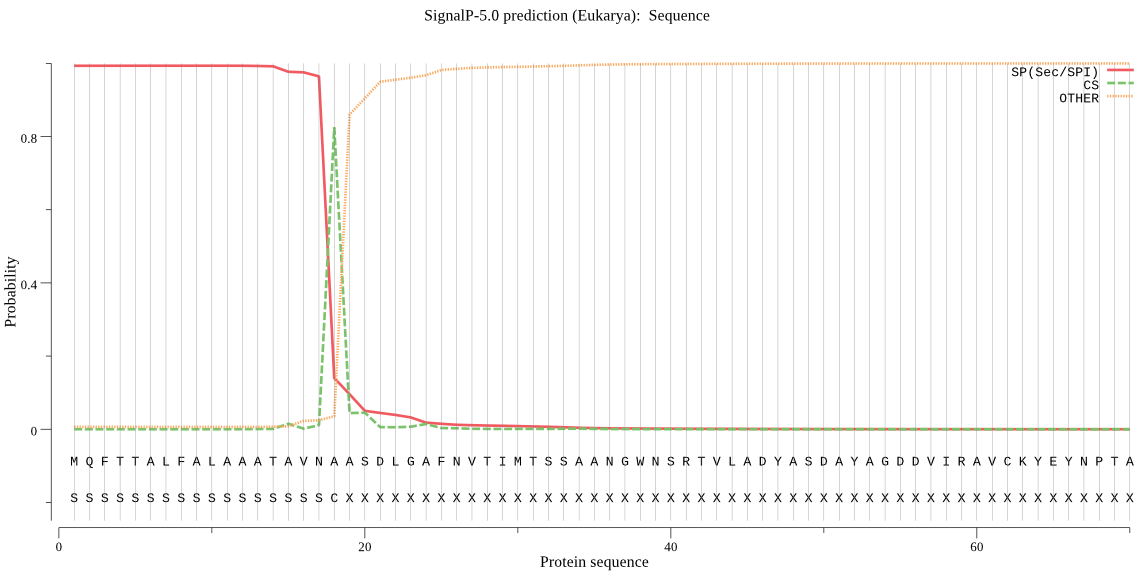


**Figure S1.** Detection of AsCEP112 Protein Signal Peptide.

# Figure S2

**Figure S2.** phylogenetic analysis of AsCEP112. Phylogenetic tree showing genetic relationships of AsCEP112 protein.

**
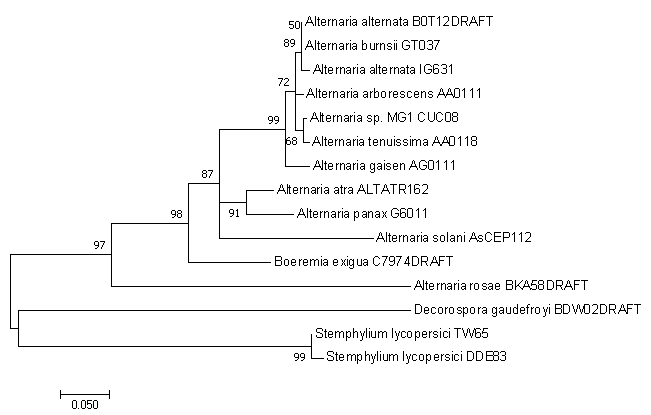
**

# Figure S3


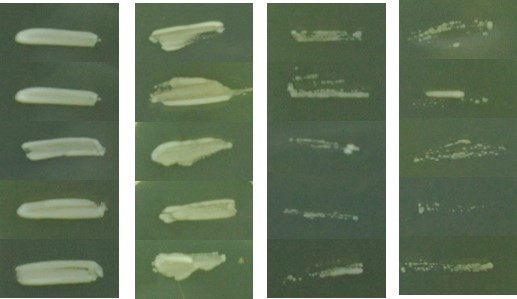

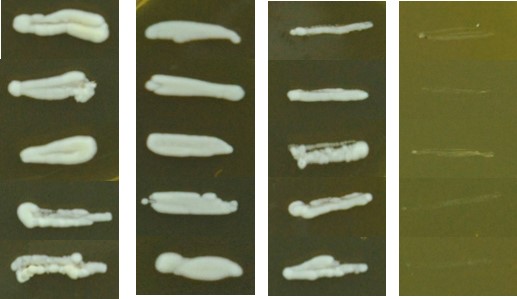


YTK12

pSUC2

pSUC-Avr1b^SP^

pSUC-AsCEP112^SP^

CMD-W YPRAA

**Figure S3**. The signal peptide (SP) of AsCEP112 is functional. The validation of the function of AsCEP112^SP^ with yeast signal trap assay. The YTK12 yeast strain containing pSUC2 is able to grow on a CMD−W medium without tryptophan, but not on YPRAA medium. AsCEP112^SP^ can grow on both CMD−W and YPRAA media. The SP of Avr1b was used as positive control.

# Figure S4

| EV | AsCEP112(FL/NSP) |
| --- | --- |
| INF1 | AsCEP112(FL/NSP)+INF1 |


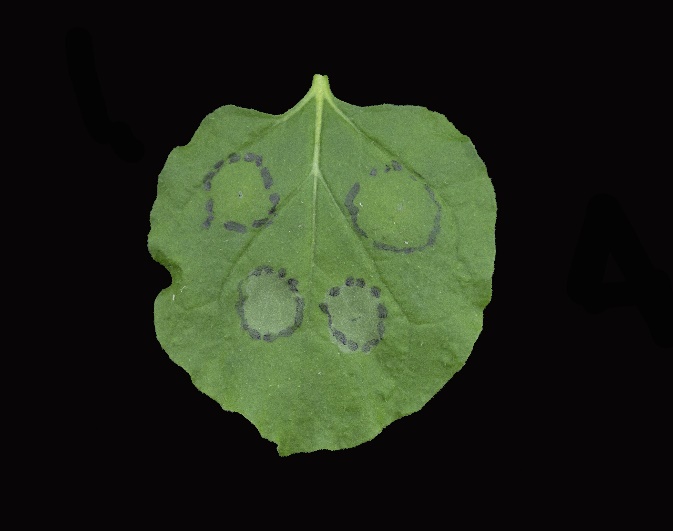


**A**


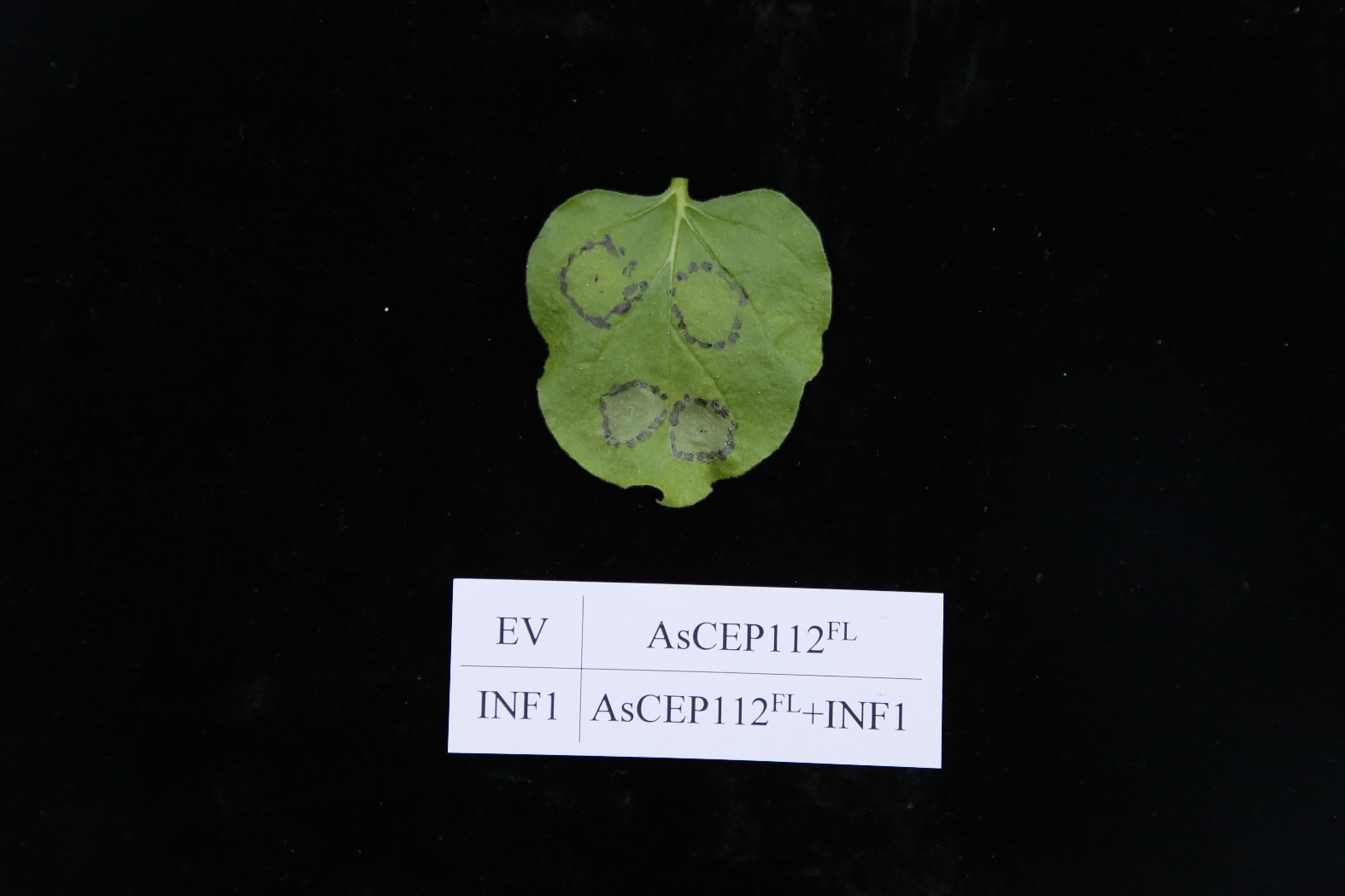


**B**

**Supplementary Figure S4.** Transient expression of *AsCEP112* in *N. benthamiana* leaves. The upper left and upper right corners of the leaf were injected with control (EV) and AsCEP112 (FL/NSP), respectively. The lower left and right corners were respectively injected with INF1 and AsCEP112 (FL/NSP) coupled with INF1.

# Figure S5


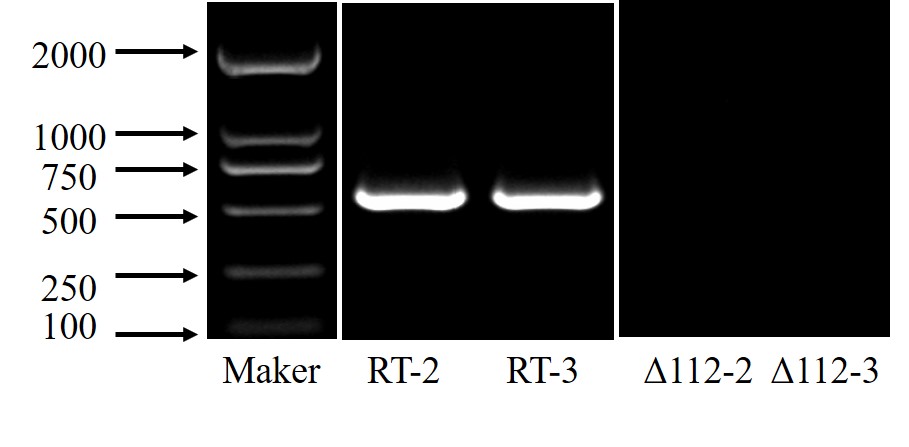


**Figure S5.** *Hyg* gene replacement and PCR screening of mutant and revertant strains. Verification of the *AsCEP112* gene in the genomes of mutant and revertant strains (RT).

# Figure S6

**Figure S6.** The colony areas and growth radii of the WT, *AsCEP112* mutant and revertant strains.


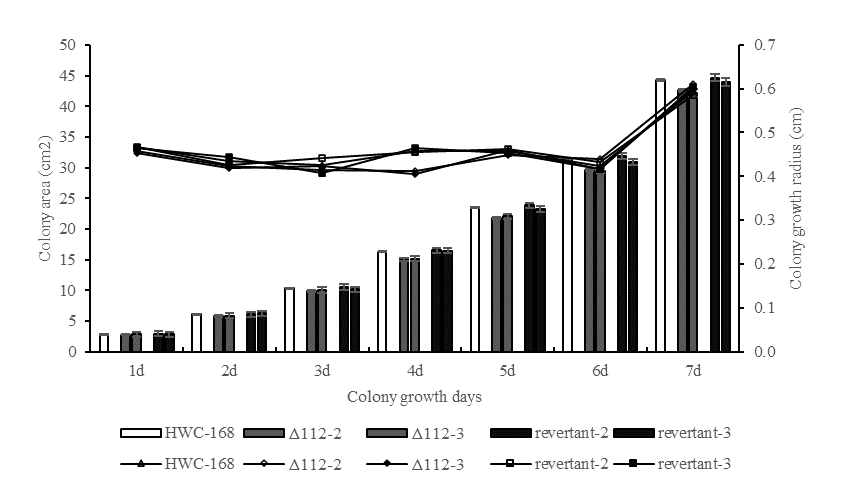


# Figure S7

HWC-168 Δ112-2 Δ112-3 revertant strain-2 revertant strain-3


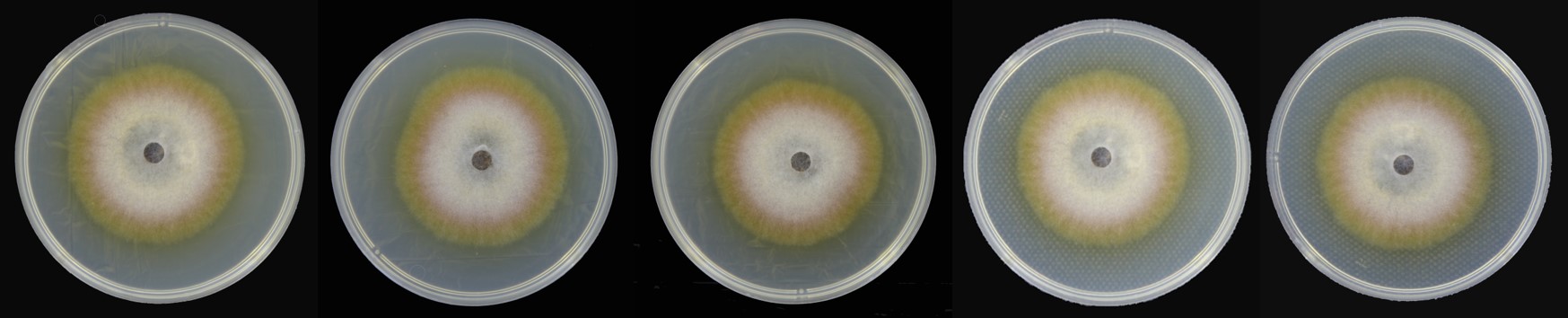


**Figure S7.** Determination of the phenotypes of the wild-type, *AsCEP112* mutant and revertant strains. The colony phenotypes of *Alternaria solani* wild-type (left 1), *AsCEP112* mutant (middle 2, 3) and revertant (right 4, 5) strains cultured on PDA medium for 7 d at 25°C in the dark.

# Figure S8

**Figure S8** Pathogenicity detection of *AsCEP112* gene. The isolated potato leaves were inoculated with the spore suspensions of AsCEP112 mutant strains (left, leaf tip to petiole direction), wild-type strains (upper right, leaf tip to petiole direction) and revertant strains (lower right, leaf tip to petiole direction).


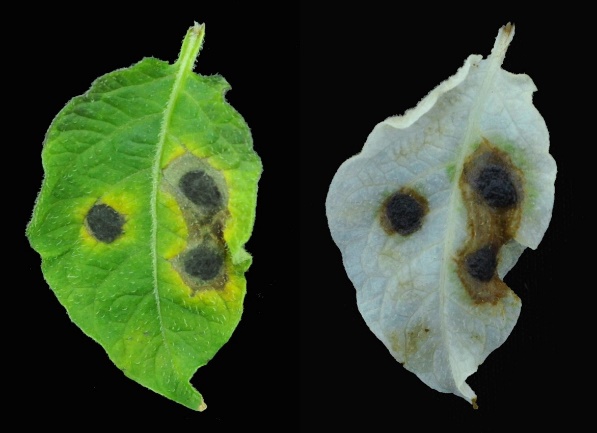


AsCEP112 DAB

AsCEP112 DAB


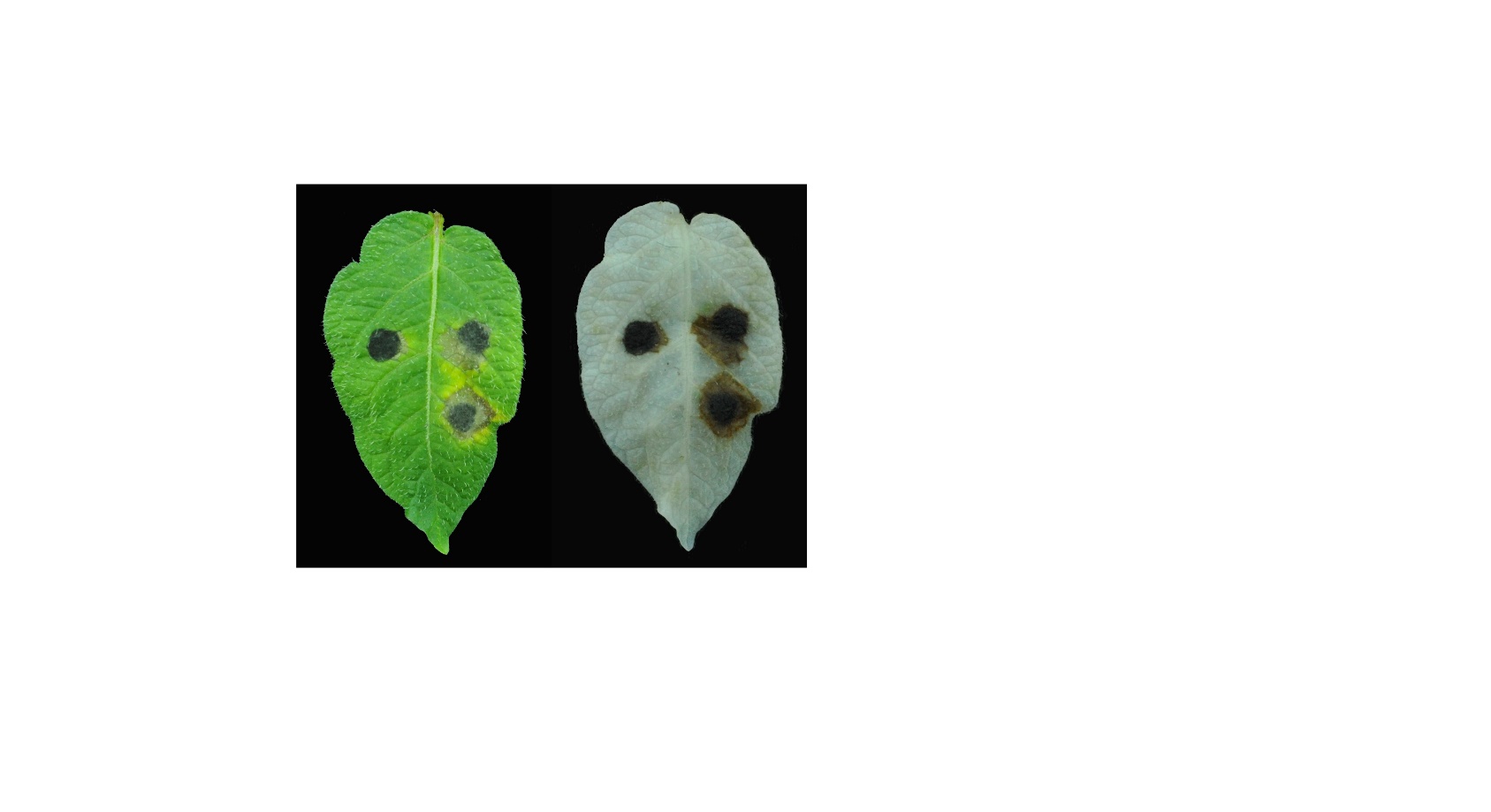


# Figure S9

**Figure S9** Full-length gel of Figure S5. The red line is the intercepted part.


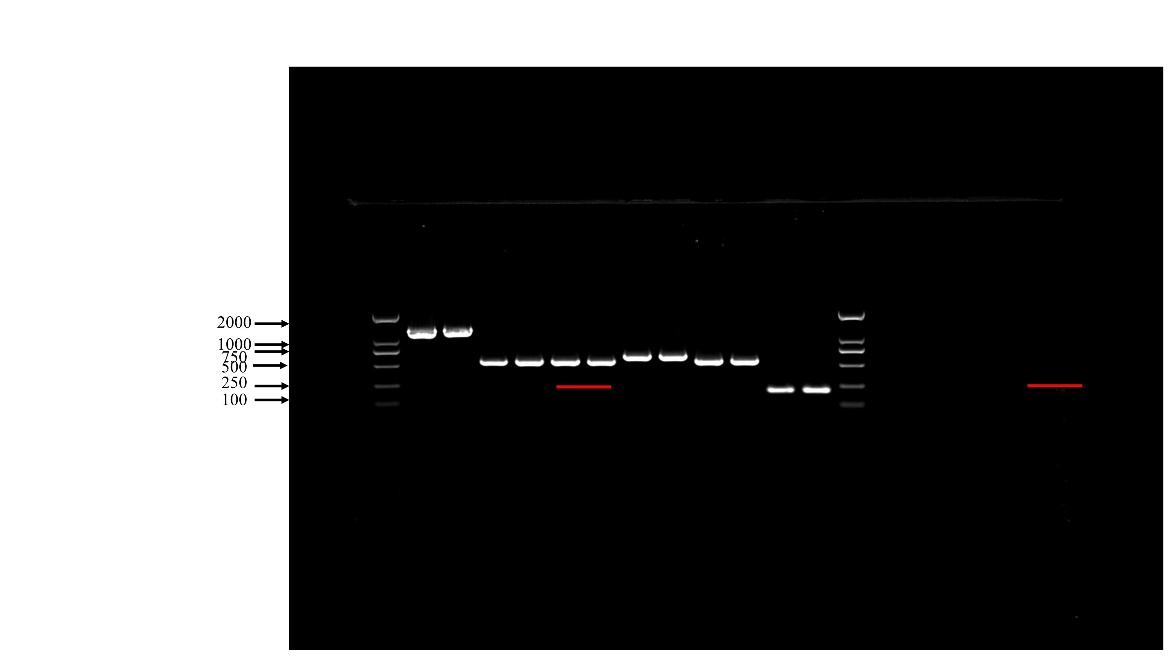


# Figure S10


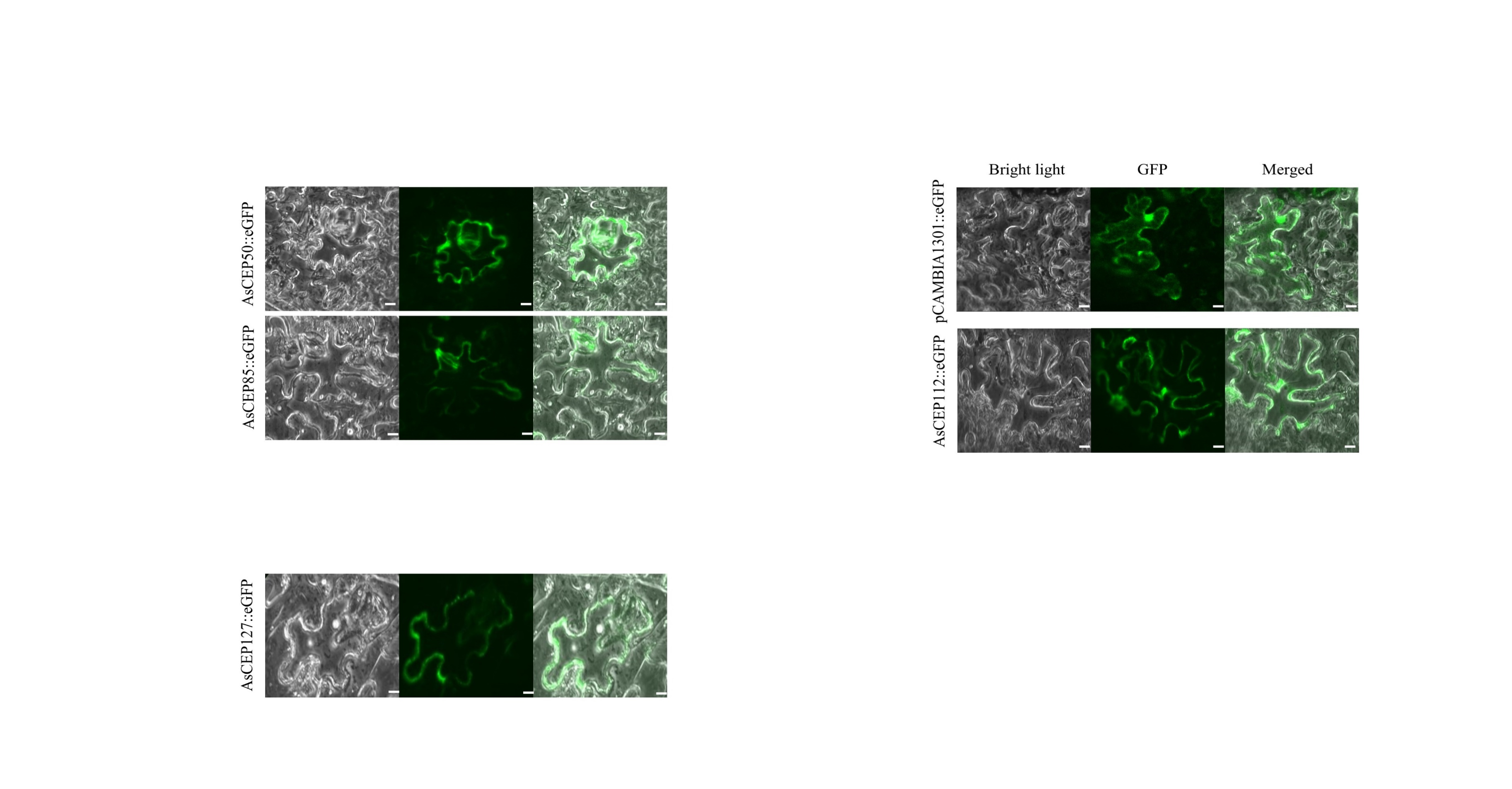


**Figure S10** Subcellular localization of *AsCEP112* proteins in *N. benthamiana*. The *Agrobacterium* strain EHA105 containing the pCAMBIA1301 vector, as the control, and the *AsCEP112* gene were independently transiently expressed in *N. benthamiana* leaves. Bar = 20 μm.

# Figure S11


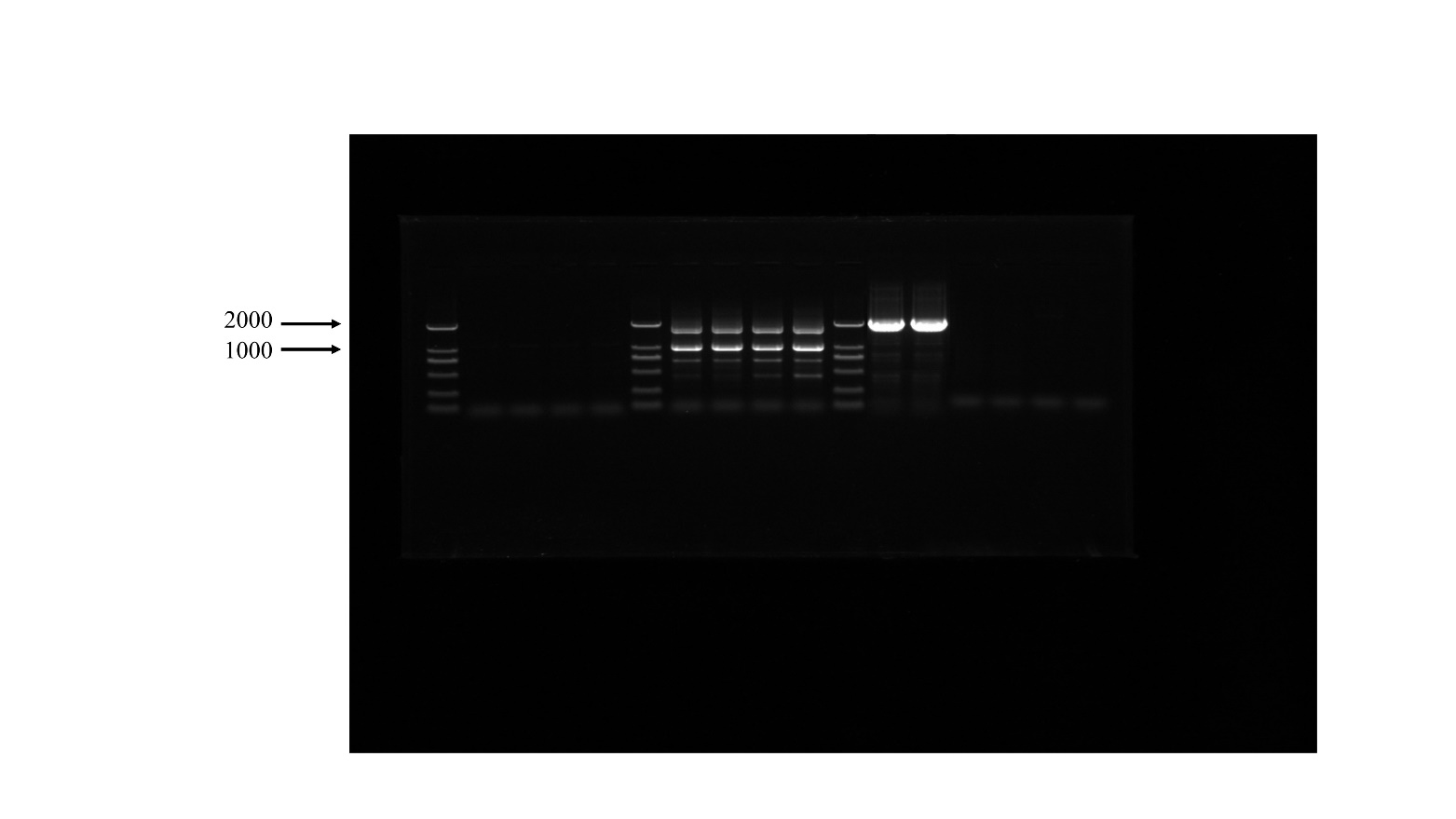


**Figure S11** Full-length gel of Figure 4B. The red box is the intercepted part.

# Figure S12

Bright light GFP Merged


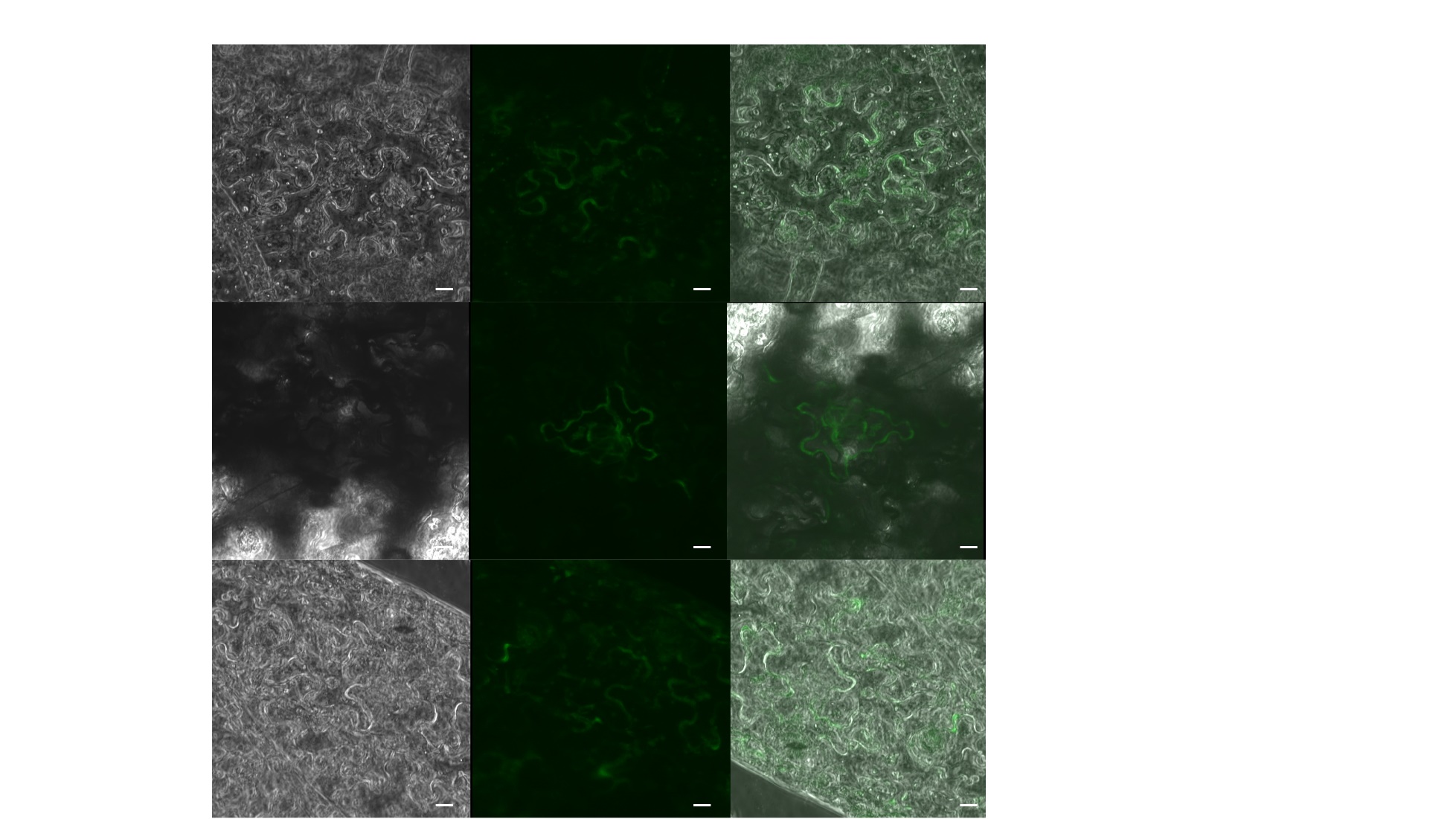


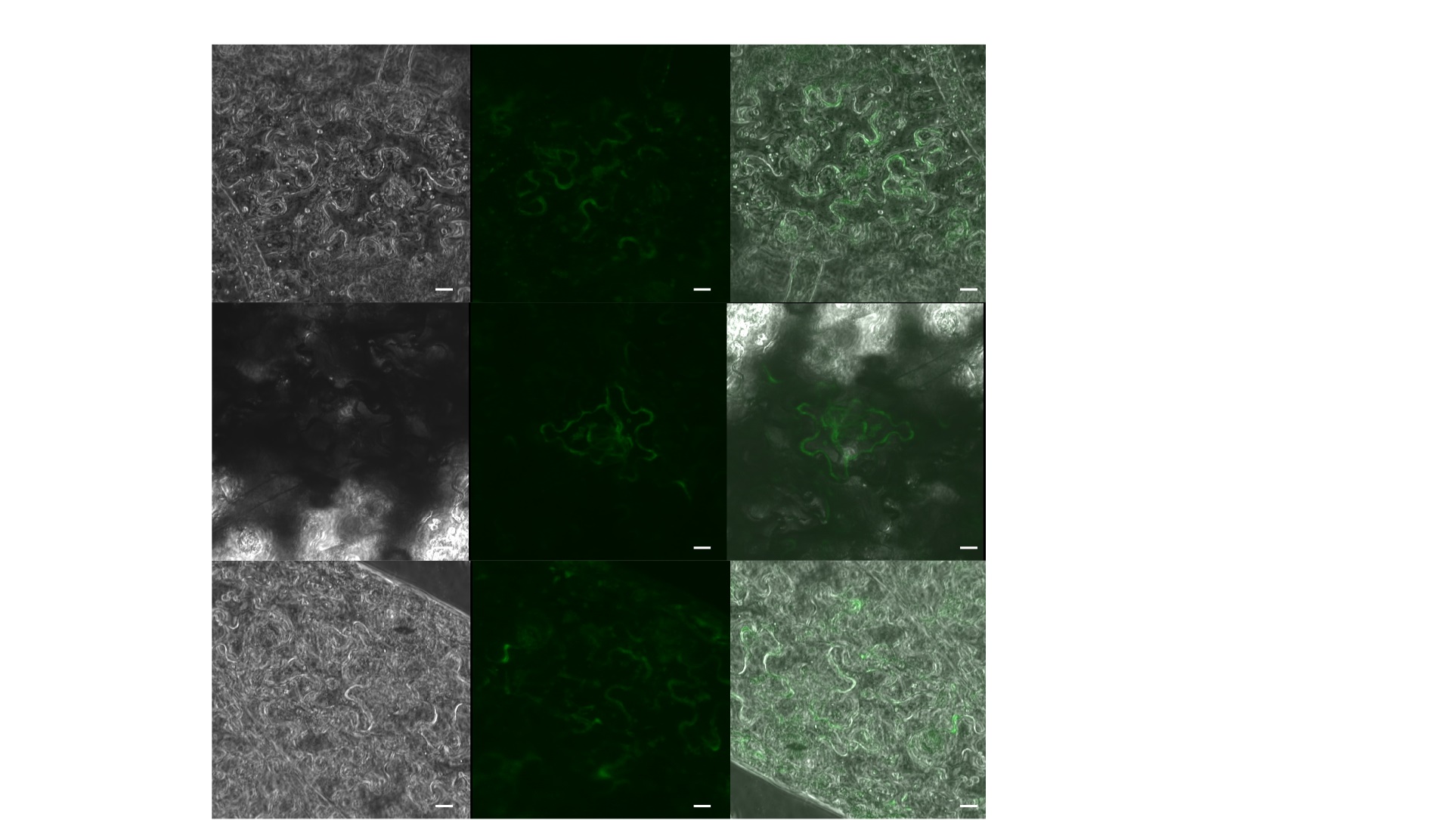


**Figure S12** Subcellular localization of *AsCEP112* proteins in *N. benthamiana*. All images are AsCEP112.
